# Supplementary material for: Single-cell multi-omics profiling links dynamic DNA methylation to cell fate decisions during mouse early organogenesis
Source: Genome Biol. 2022 Sep 26;23:202. doi: 10.1186/s13059-022-02762-3 (PMC9511790; doi:10.1186/s13059-022-02762-3)
Supplement: Supplementary file 1 — Additional file 1: Figure S1. General statistics and quality control metrics for Dnmt1-/-, Dnmt3a-/-, Dnmt3b-/- and WT scRNA-seq libraries. Figure S2. Cell type assignments for Dnmt1-/-, Dnmt3a-/-, Dnmt3b-/- and WT embryos. Figure S3. Inference of embryonic stage for Dnmt1-/-, Dnmt3a-/- and Dnmt3b-/- embryos. Figure S4. Expression changes of imprinted genes in Dnmt1-/-, Dnmt3a-/- and Dnmt3b-/- embryos. Figure S5. Expression changes of germline genes in Dnmt1-/-, Dnmt3a-/- and Dnmt3b-/- embryos. Figure S6. Expression changes of repetitive elements in Dnmt1-/-, Dnmt3a-/- and Dnmt3b-/- embryos. Figure S7. Comparison of differential expression changes with a published bulk RNA-seq study. Figure S8. Overview of the Tet-TKO chimaera assay. Figure S9. Mapping, cell type assignments and embryo staging for Tet-TKO scRNA-seq samples. Figure S10. Differential gene expression analysis between WT and Tet-TKO embryos. Figure S11. Quality control (QC) metrics for scNMT-seq Tet-TKO embryos. Figure S12. Cell type assignments of WT and Tet-TKO scNMT-seq cells. Figure S13. DNA methylation and chromatin accessibility at promoters and lineage-specific enhancers for different cell types in the Tet-TKO scNMT-seq experiment. Figure S14. Examples of individual cis-regulatory regions that are dsyregulated in Tet-TKO cells. [file 13059_2022_2762_MOESM1_ESM.pdf]

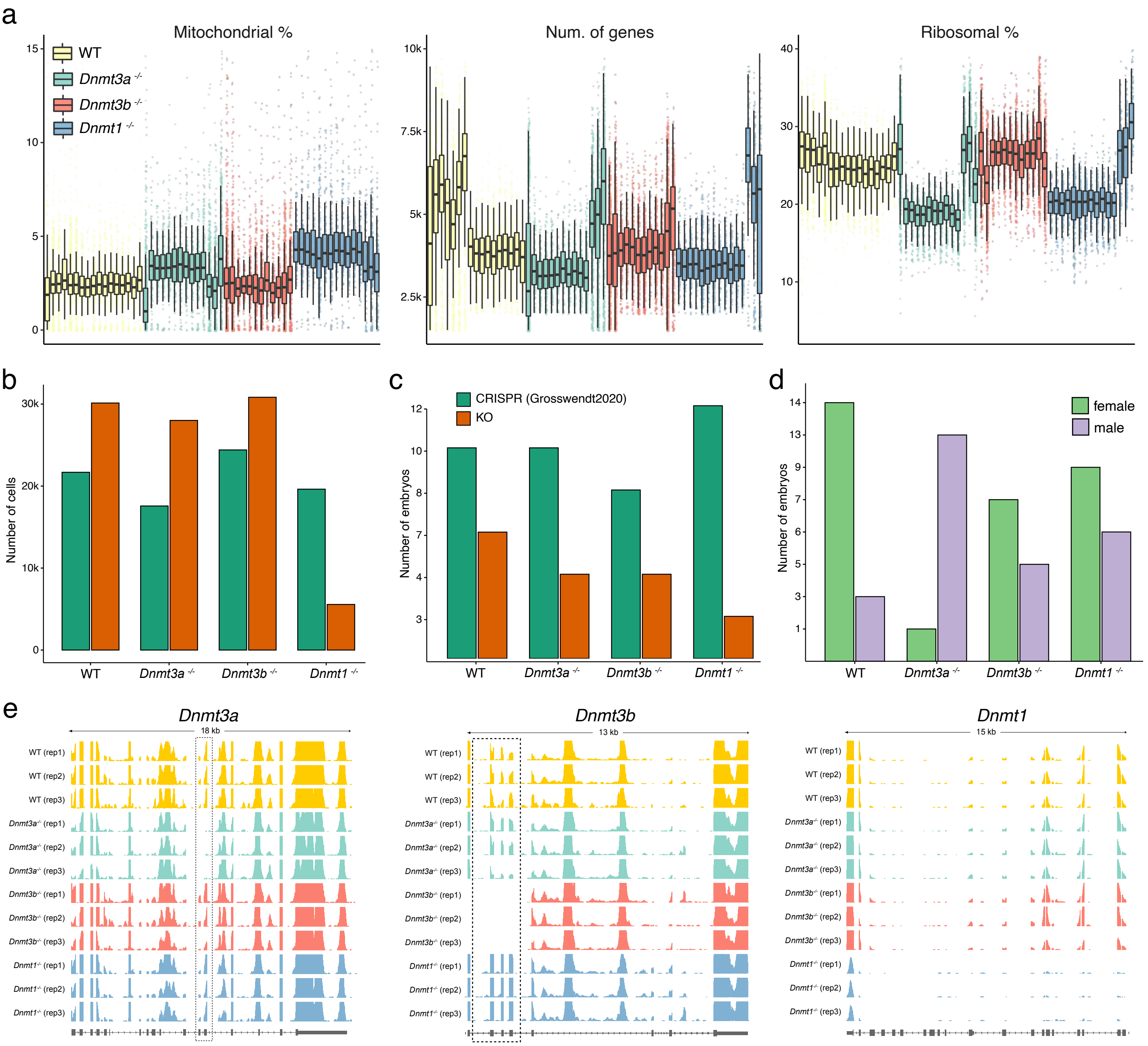

Figure S1: General statistics and quality control metrics for *Dnmt1*$^{-/-}$, *Dnmt3a*$^{-/-}$, *Dnmt3b*$^{-/-}$ and WT scRNA-seq libraries.

(a) Percentage of reads mapping to mitochondria (left) and ribosomal genes (right). The middle plot displays the number of genes detected per cell (> 0 counts). Each data point is a single-cell and each box is a sample, coloured by genotype. Shown are cells which pass QC metrics only.

(b) Number of cells analysed in this study, split by data set of origin. Orange bar plots display the number of cells from KO embryos profiled in this study together. Green bar plots display the number of cells from a published dataset where the same enzymes were disrupted using zygotic CRISPR-Cas9 injection [23].

(c) Number of embryos analysed in this study, split by data set of origin, as in (b).

(d) Number of male and female embryos profiled per genotype. The sex of the embryo was computationally inferred from the fraction of reads mapping to the Y chromosome.

(e) Genome browser snapshots displaying transcriptomic coverage. Each track displays a representative sample in which all cells are pooled. Shown are the regions of the *Dnmt3a* (top), *Dnmt3b* (middle) and *Dnmt1* (bottom) gene where deletions are expected. Dashed-line boxes indicate the exons that are deleted in each gene. Snapshots are generated using the IGV browser [59].

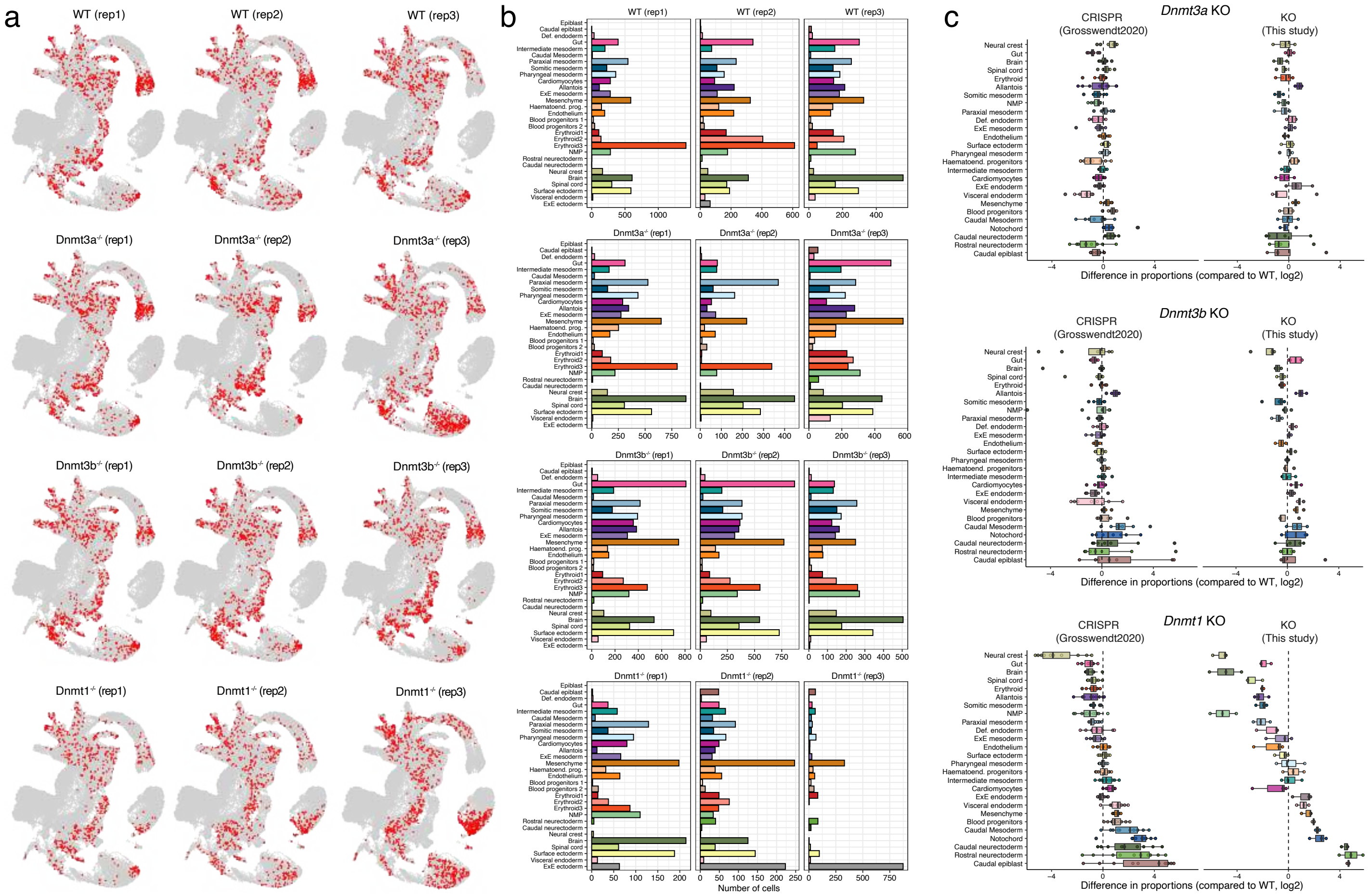

Figure S2: Celltype assignments for *Dnmt1*$^{-/-}$, *Dnmt3a*$^{-/-}$, *Dnmt3b*$^{-/-}$ and WT embryos.

(a) Mapping of the KO cells to the reference atlas using the matching nearest neighbours (MNN) algorithm [26]. Each plot shows the UMAP of the reference atlas [25] with nearest neighbours cells coloured in red. Each plot shows a different embryo. Four representative samples are shown for each genotype.

(b) Bar plots display the number of cells for each embryo and cell type.

(c) Boxplots displaying the difference in cell type proportions between knockout and wildtype from the two different datasets: CRISPR [23] and KO (this study). The number of cells and embryos for each data set is shown in Figure 1a.

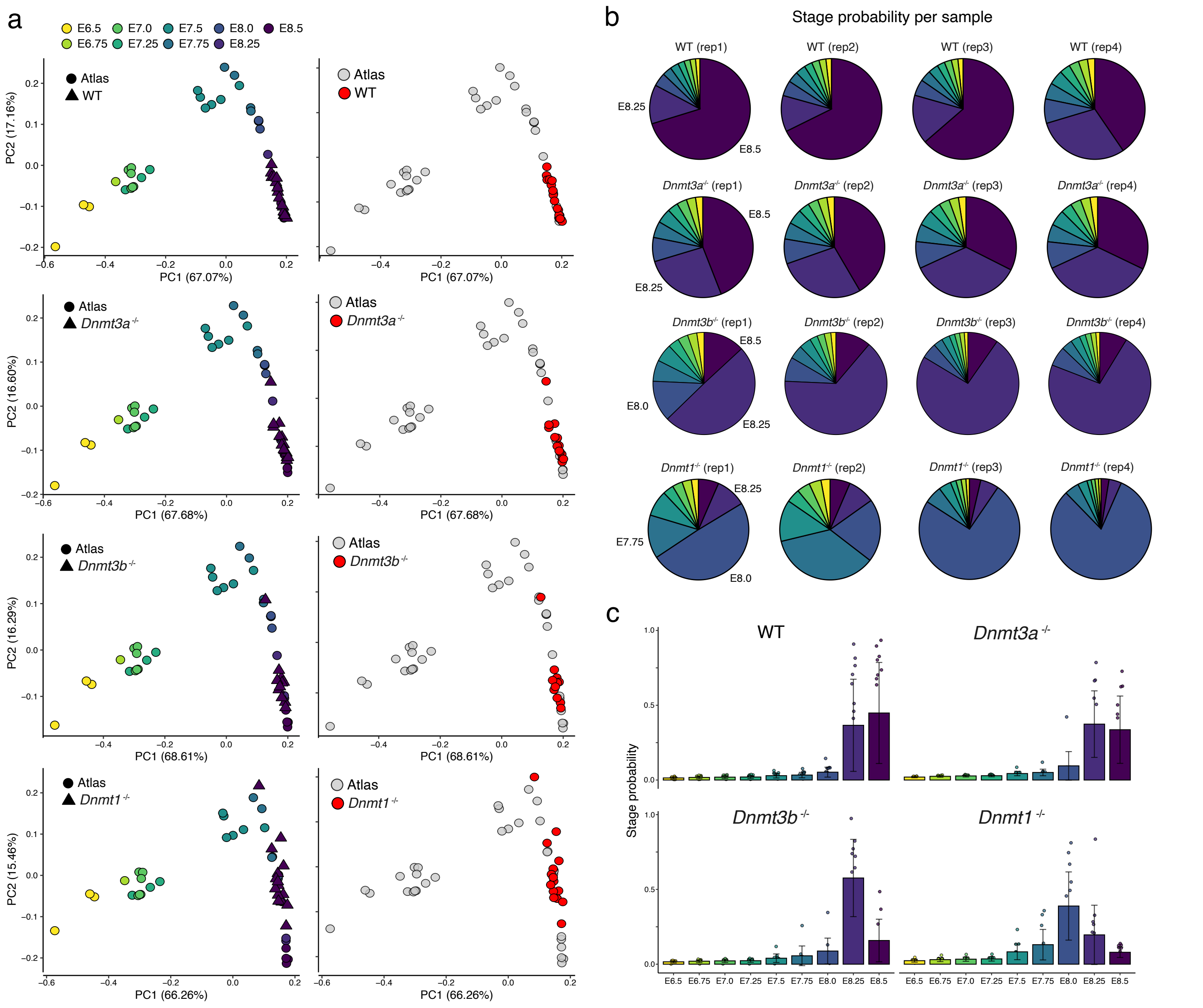

Figure S3: Inference of embryonic stage for *Dnmt1*$^{-/-}$, *Dnmt3a*$^{-/-}$ and *Dnmt3b*$^{-/-}$ embryos.

(a) Scatter plot of PCA values computed from the cell type distributions, where each dot corresponds to a different sample. Left: Triangles are embryos profiled in this study, circles are embryos from the wildtype reference atlas. Reference embryos are coloured by their stage. Right: as left except embryos are coloured by the dataset (reference atlas versus this study).

(b) Pie charts display the probability that a given embryo is mapped to a given stage. Four representative samples are selected for each genotype.

(c) Bar plots show the average probability that a given embryo is mapped to a given stage, with the standard deviation shown as whiskers. Each data point is a single embryo and each genotype is plotted separately.

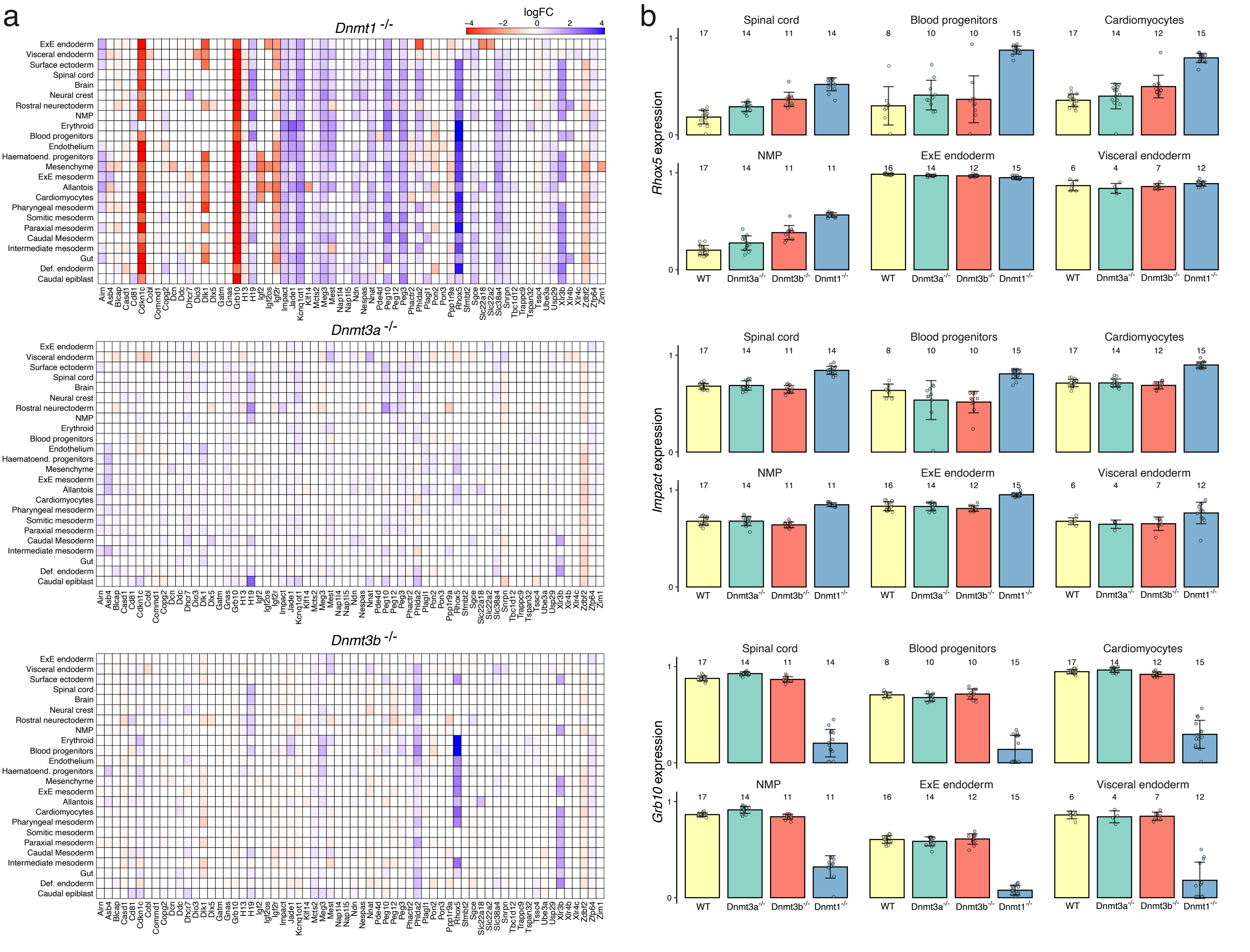

Figure S4: Expression changes of imprinted genes in *Dnmt1*$^{-/-}$, *Dnmt3a*$^{-/-}$ and *Dnmt3b*$^{-/-}$ embryos.

(a) Heatmaps display the log fold change in gene expression between Dnmt mutants and WT.

(b) Gene expression levels in pseudobulked samples for each genotype. Each data point corresponds to a different embryo and cell type.

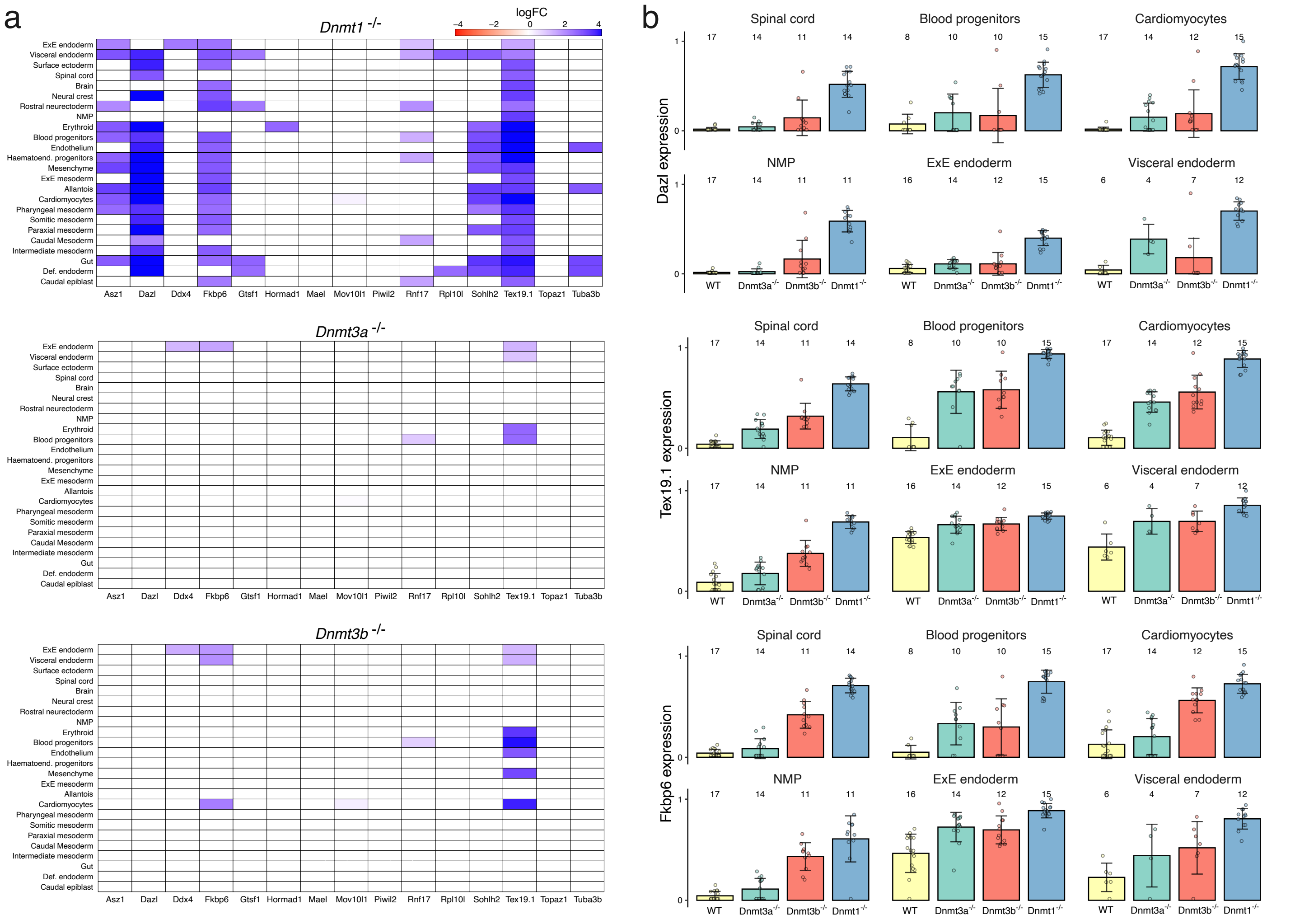

Figure S5: Expression changes of germline genes in *Dnmt1*$^{-/-}$, *Dnmt3a*$^{-/-}$ and *Dnmt3b*$^{-/-}$ embryos.

(a) Heatmaps display the log fold change in gene expression between Dnmt mutants and WT.

(b) Gene expression levels in pseudobulked samples for each genotype. Each data point corresponds to a different embryo and cell type.

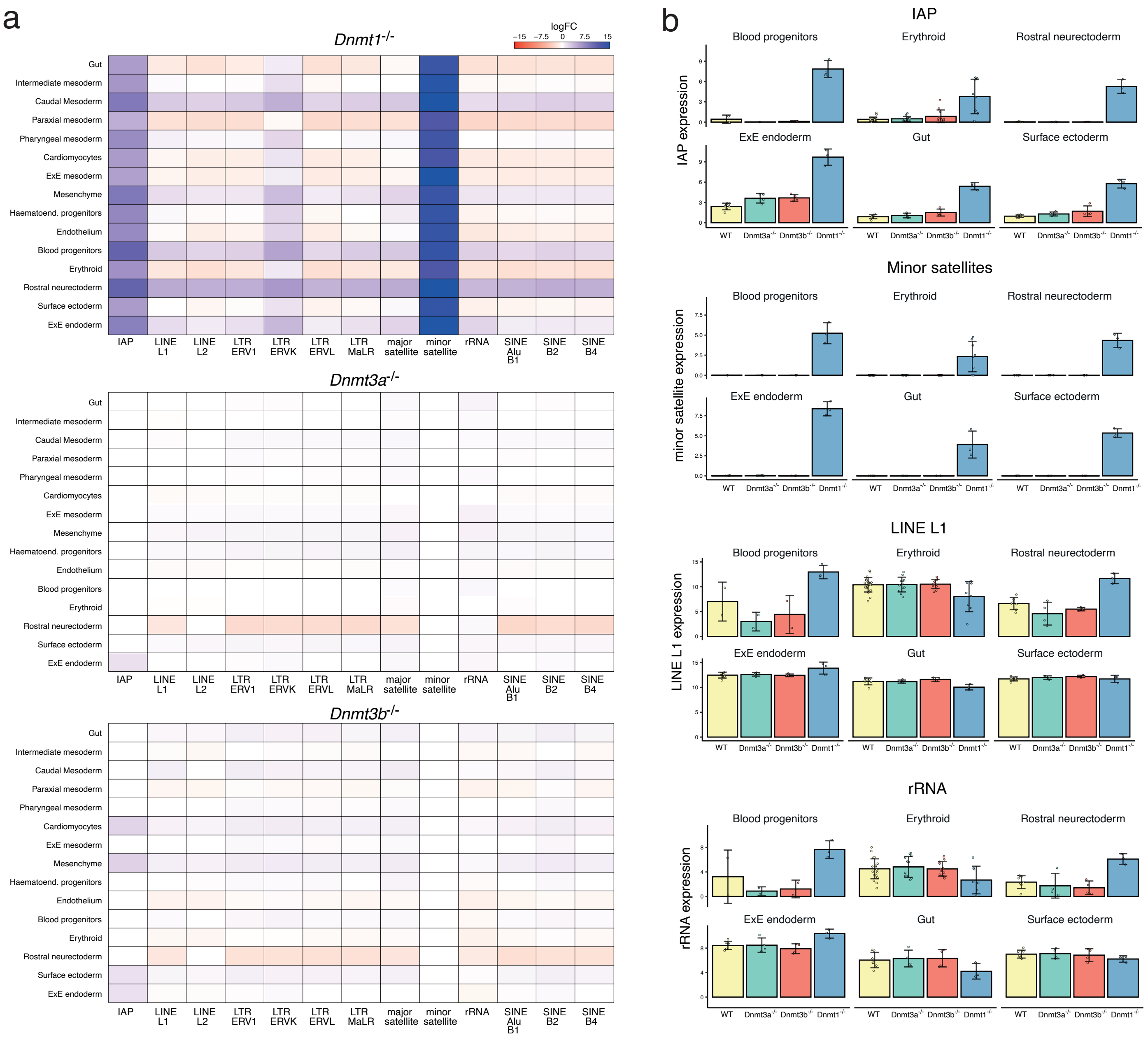

Figure S6: Expression changes of repetitive elements in *Dnmt1*$^{-/-}$, *Dnmt3a*$^{-/-}$ and *Dnmt3b*$^{-/-}$ embryos.

(a) Heatmaps display the log fold change in RNA expression of repetitive elements between Dnmt mutants and WT. Shown are different classes of repetitive elements. Note that the difficult mappability and low expression levels of repetitive elements makes it challenging to measure the expression of individual elements in single cells. Here we quantified RNA expression of repetitive elements after (1) aggregating the reads across all individual elements from the same type and (2) aggregating the reads across all cells from a single embryo that are annotated to the same cell type. Thus, in contrast to previous studies that employed bulk RNA-seq, this approach enables us to interrogate celltype-specific changes in the expression of repetitive elements.

(b) Gene expression levels in pseudobulked samples for each genotype. Each data point corresponds to a different embryo and cell type.

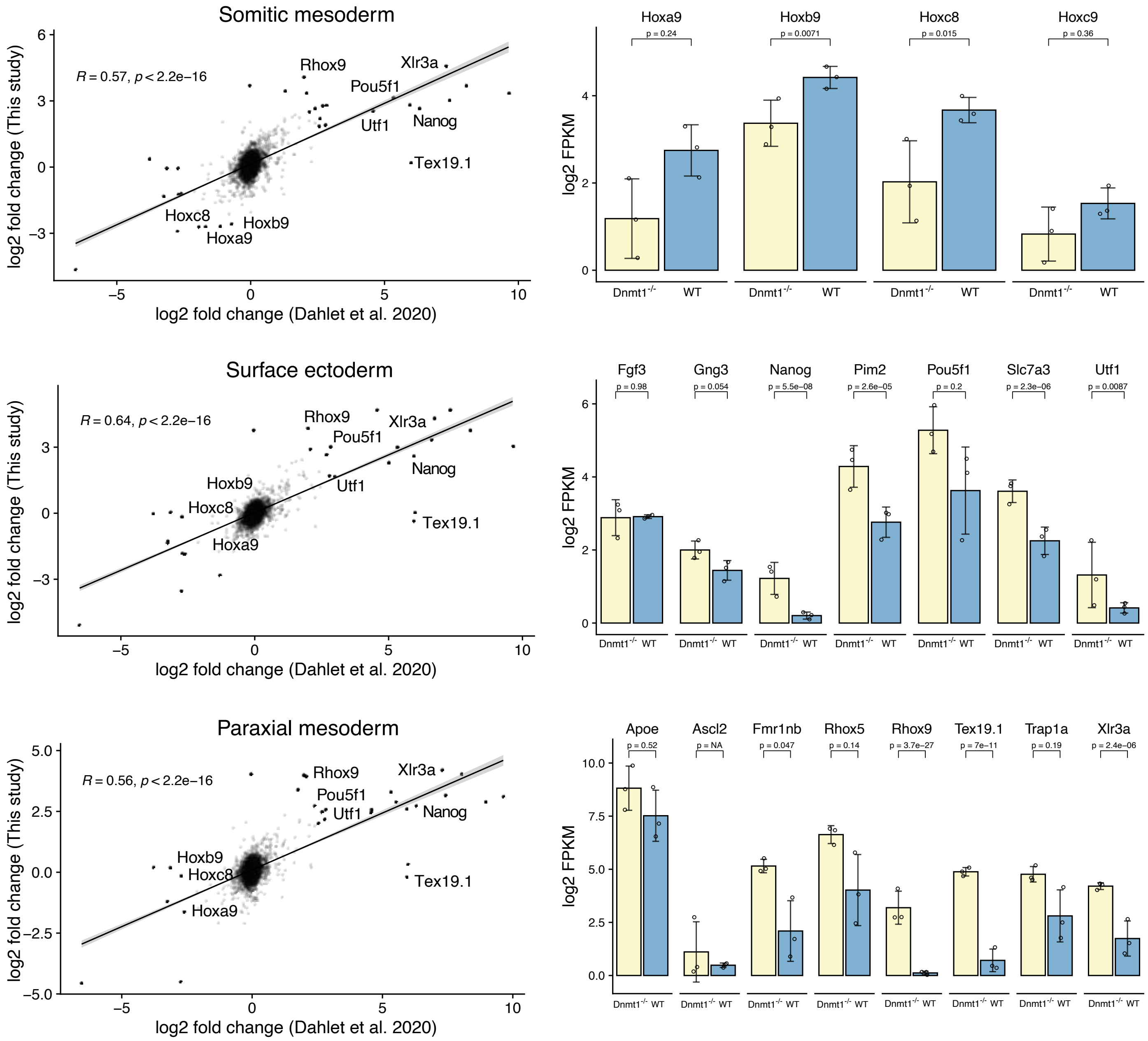

Figure S7: Comparison of differential expression changes with a published bulk RNA-seq study.

(a) Scatterplots showing the log2 fold-change in gene expression between WT and *Dnmt1*$^{-/-}$ embryos from published bulk RNA-seq data [22] (x-axis) and this study (y-axis). Each dot is a gene. Some Hox genes, pluripotency markers and extra-embryonic celltype markers are highlighted (i.e. the same genes shown in Figure 2). Note that our analysis was performed separately for each celltype whereas the bulk analysis was only performed once, hence the x-axis values will be the same for each sub-plot.

(b) Barplots displaying gene expression values from published bulk data [22] in WT and *Dnmt1*$^{-/-}$ embryos. Shown are genes featured in Figure 2 which are differentially expressed in at least one cell type in our analysis.

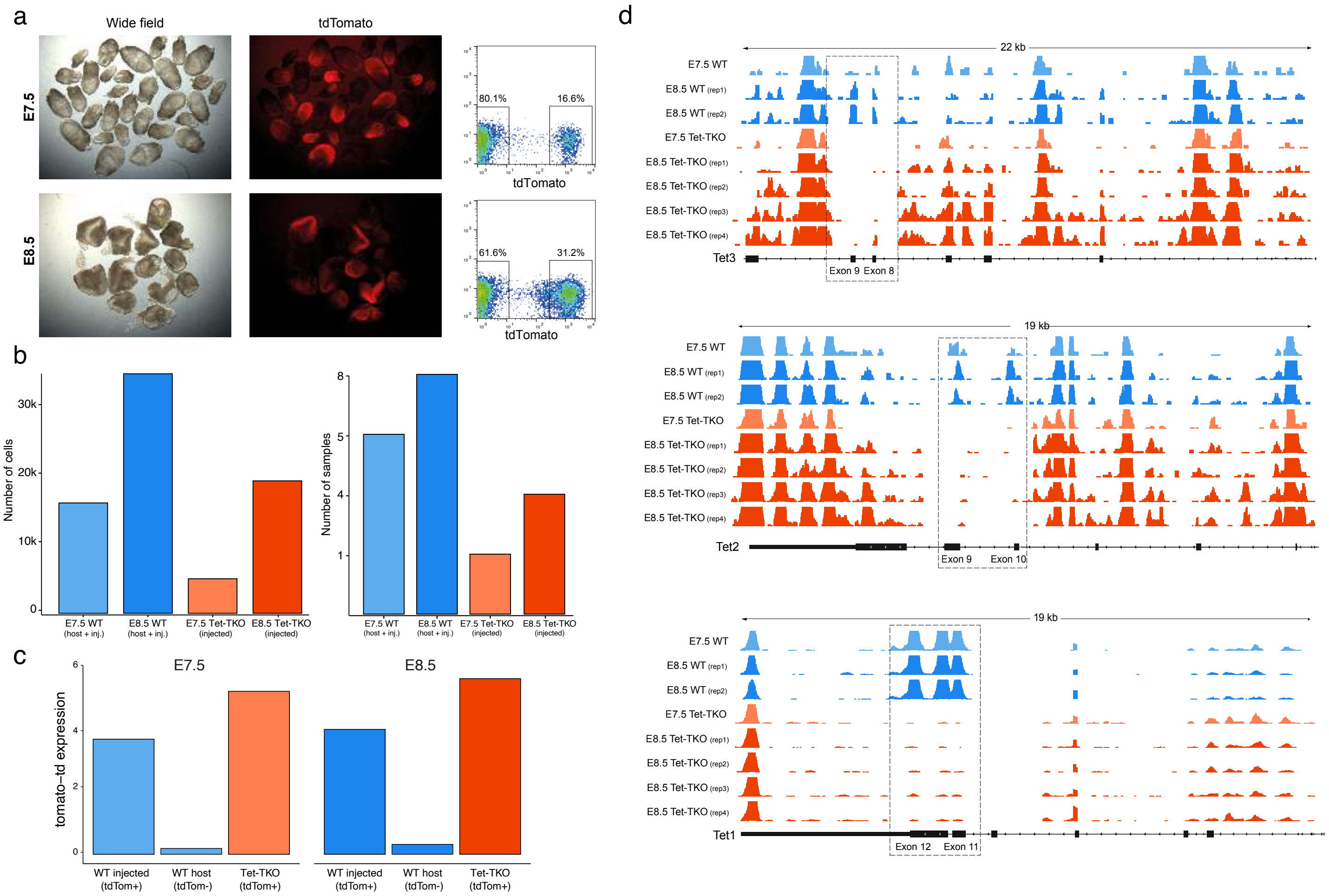

Figure S8: Overview of the *Tet*-TKO chimaera assay.

(a) Images of *Tet*-TKO chimeric embryos at E7.5 and E8.5 (left: brightfield; middle: tdTomato fluorescence). Right, flow cytometry plots showing tdTomato fluorescence levels and sorting gates used.

(b) Number of cells (left) and number of samples (right) profiled for each stage and sample type after quality control filtering. Note that two groups of WT cells exist: WT injected cells (TdTomato+) and WT host cells (TdTomato-).

(c) Barplots displaying tdTomato expression levels (log2 normalised reads) by sample type. The expression values of all cells from a given sample type are pooled together.

(d) Genome browser plots displaying the distributions of RNA-seq reads mapping to the three Tet genes for each sample profiled (single cells are merged per sample). Dashed-line boxes indicate the exons that were deleted in the *Tet*-TKO cell line.

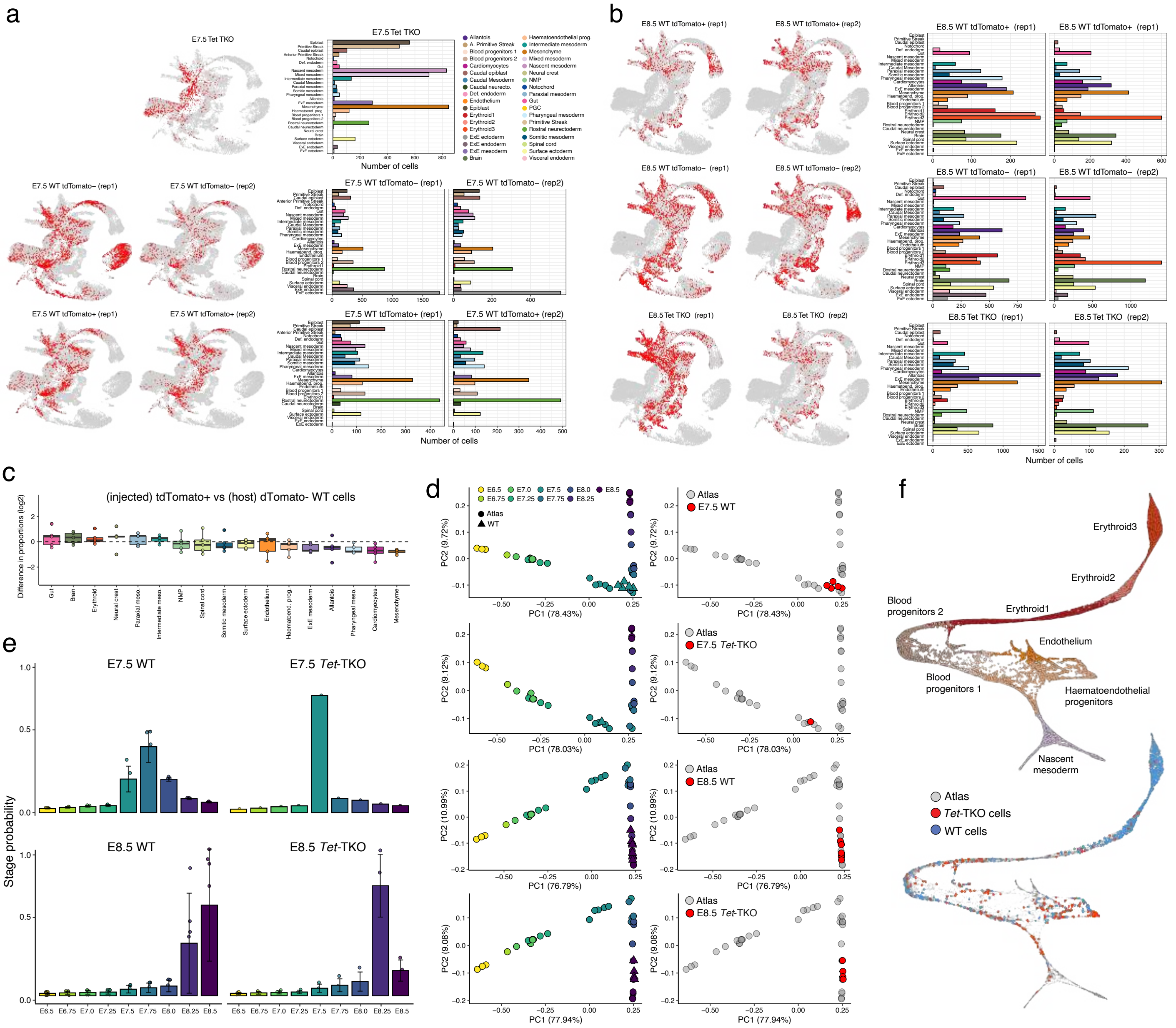

Figure S9: Mapping, celltype assignments and embryo staging for *Tet*-TKO scRNA-seq samples.

(a) Left: UMAP representation of the reference data set [25]. Cells coloured red are nearest neighbours to a cell in our E7.5 scRNA-seq data. Each plot corresponds to a different sample. Right: for the same samples, the bar plots display the number of cells assigned to each cell type.

(b) Left: UMAP representation of the reference data set [25]. Cells coloured red are nearest neighbours to a cell in our E8.5 scRNA-seq data. Each plot corresponds to a different sample. Right: for the same samples, the bar plots display the number of cells assigned to each cell type.

(c) Cell type abundance comparison between WT injected cells (TdTomato+) and WT host cells (TdTomato-) from the same embryos.

(d) Scatter plot of PCA values computed from the cell type distributions, where each dot corresponds to a different sample. Left: Triangles are embryos profiled in this study, circles are embryos from the wildtype reference atlas. Reference embryos are coloured by their stage. Right: as left except embryos are coloured by the dataset (reference atlas versus this study).

(e) Bar plots show the average probability that a given embryo is mapped to a given stage, with the standard deviation shown as whiskers. Each data point is a single sample and each genotype is plotted separately.

(f) Haematoendothelial differentiation trajectory from the reference dataset [25]. In the top panel cells are coloured by cell type. In the bottom panel cells that are nearest neighbours of the *Tet*-TKO scRNA-seq cells are coloured in blue (WT cells) or red (*Tet*-TKO cells)

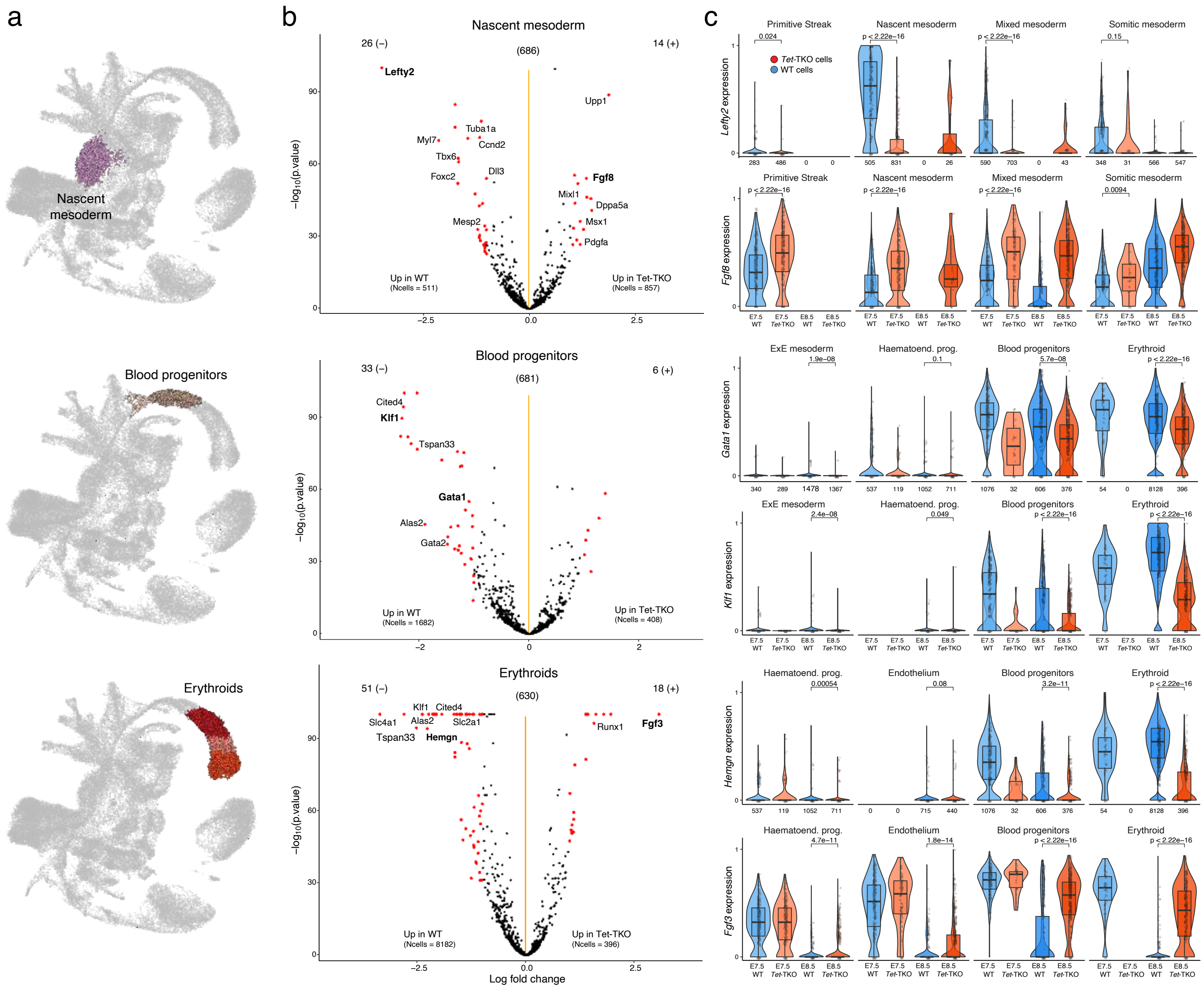

Figure S10: Differential gene expression analysis between WT and *Tet*-TKO embryos

(a) UMAP from the reference data set [25], highlighting the cell type that was used for differential expression analysis in (b).

(b) Volcano plots display the difference in gene expression levels between *Tet*-TKO and WT (x-axis) against the corresponding FDR-adjusted p-value for Nascent mesoderm (top), Blood progenitors (middle) and Erythroid (bottom) cell types. Coloured in red are genes which pass the significance threshold.

(c) Box and violin plots display the RNA expression levels (log2 normalised counts) of two representative differentially expressed genes for each of the three cell types. For each gene, its expression is shown for four selected cell types.

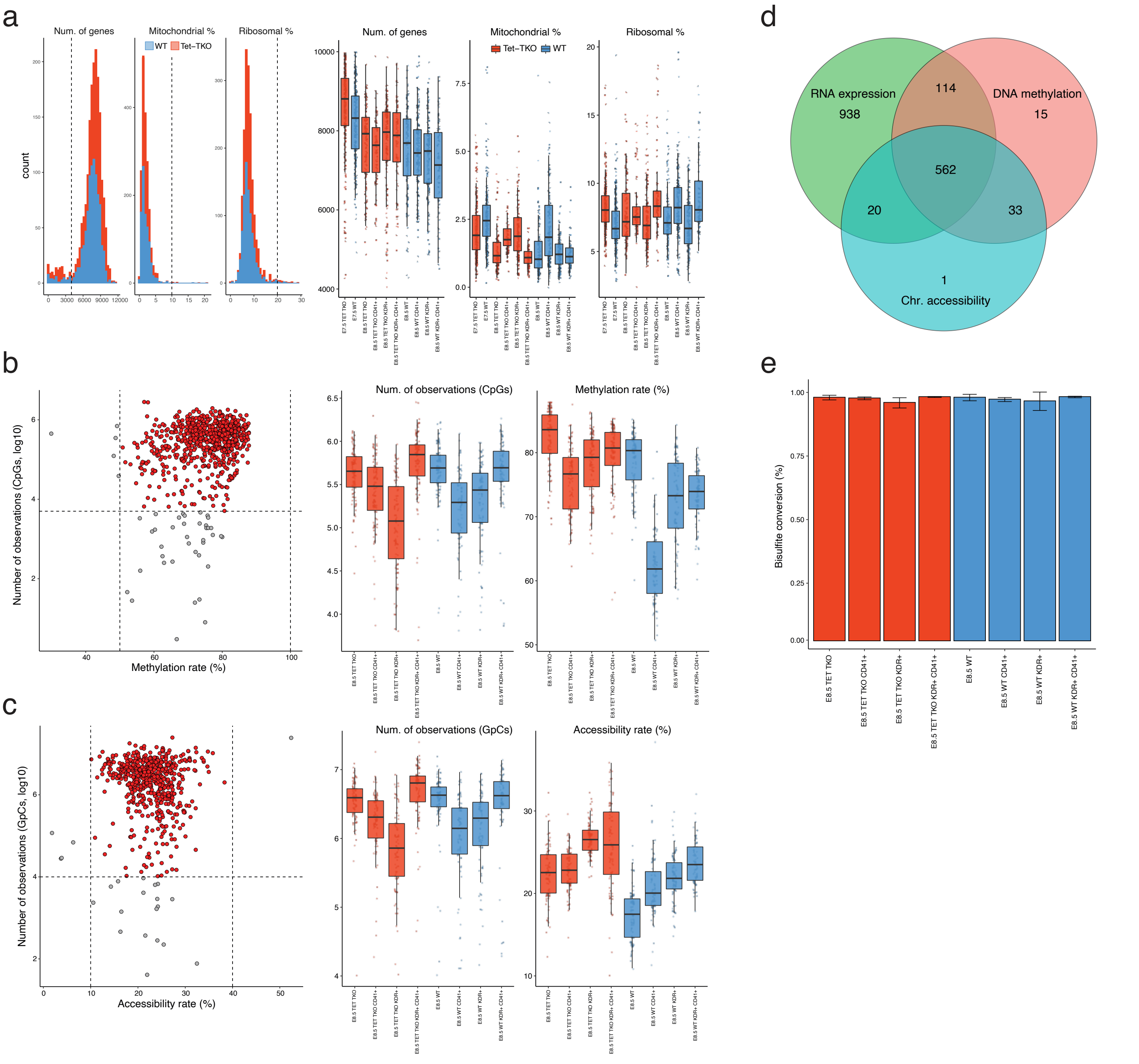

Figure S11: Quality control (QC) metrics for scNMT-seq *Tet*-TKO embryos.

(a) scRNA-seq QC metrics. Left: histograms showing the distributions of (i) number of detected genes per cell (ii) percentage of reads mapping to the mitochondrial chromosome per cell (iii) percentage of reads mapping to ribosomal genes per cell. Right: boxplots showing the same statistics as left but shown separately for each sample.

(b) Methylation QC metrics. Left: scatter plot comparing the global CpG methylation rate (x-axis, i.e. mean methylation across all CpGs in a given cell) to the CpG coverage per cell (y-axis). High quality cells are expected to have a large number of observed CpGs and the global rate to be ≥50%. Right: boxplots showing the same statistics as left but shown separately for each sample.

(c) Accessibility QC metrics. Left: scatter plot comparing the global GpC accessibility rate (x-axis, i.e. mean accessibility across all GpCs in a given cell) to the GpC coverage per cell (y-axis). High quality cells are expected to have a large number of observed GpCs and the global rate to be ≥10% and ≤40%. Right: boxplots showing the same statistics as left but shown separately for each sample.

(d) Number of cells that pass QC for each data modality. Note that we sequenced 768 cells using scNMT-seq (three data modalities) together with an additional 1056 cells using only scRNA-seq. The increased sample size of scRNA-seq data was used to aid cell type annotation. A total of N=562 cells passed QC for all three data modalities.

(e) Bisulfite conversion rates (%) for each sample. Each dot corresponds to an individual cell.

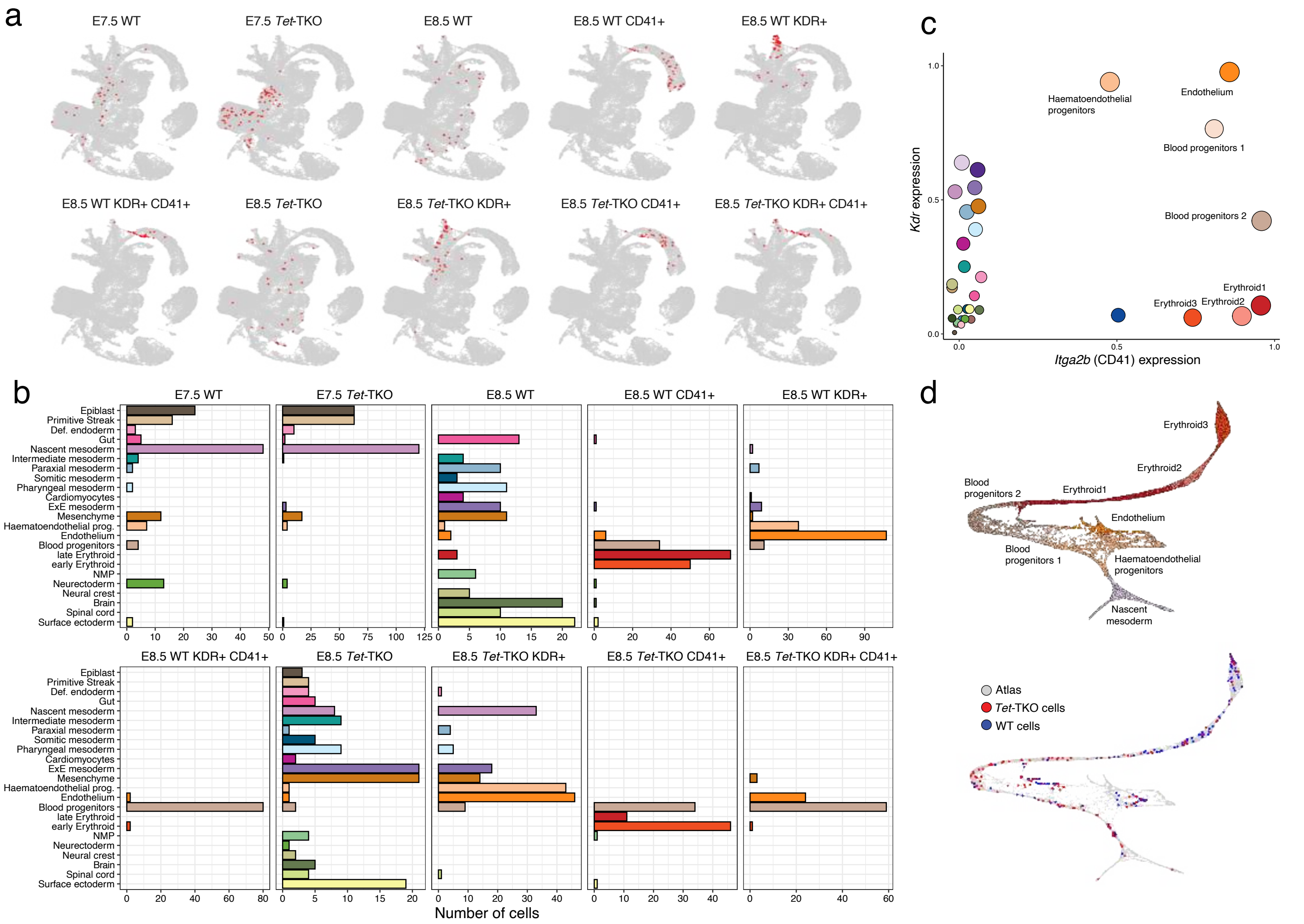

Figure S12: Celltype assignments of WT and *Tet*-TKO scNMT-seq cells

(a) Dimensionality reduction of gene expression (UMAP) from [25]. Cells coloured red are nearest neighbours to a cell in our scNMT-seq data. Each plot shows a different stage and sorting strategy.

(b) Barplots showing cell type assignments split by stage and sorting strategy.

(c) Expression levels of the markers used to sort cells in the wildtype reference dataset [25]. Each datapoint is a pseudobulked celltype.

(d) Haematoendothelial differentiation trajectory from the reference dataset [25]. In the top panel cells are coloured by cell type. In the bottom panel cells that are nearest neighbours of our scNMT-seq data are coloured in blue (WT cells) or red (*Tet*-TKO cells)

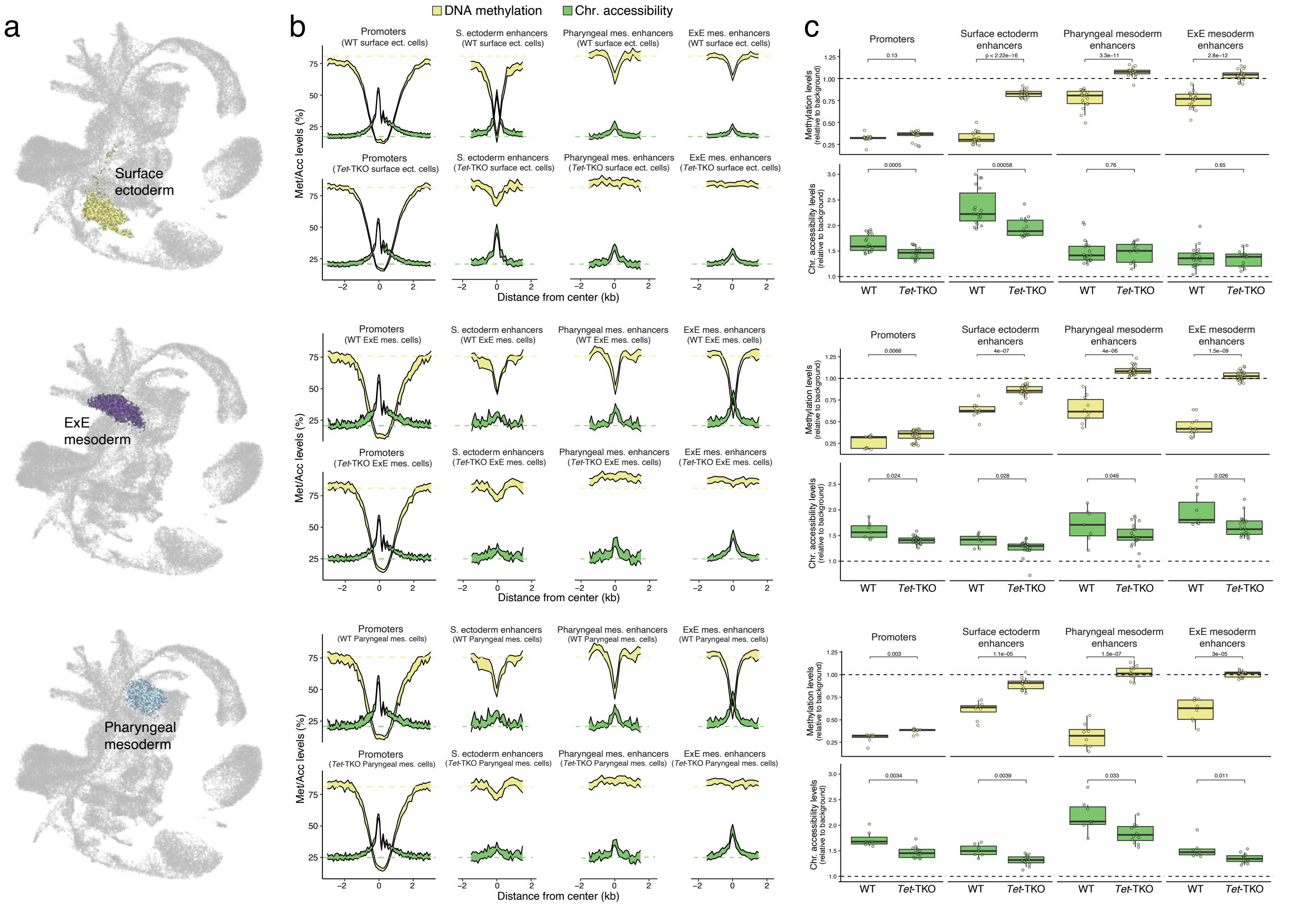

Figure S13: DNA methylation and chromatin accessibility at promoters and lineage-specific enhancers for different cell types in the *Tet*-TKO scNMT-seq experiment.

(a) UMAP from the reference data set [25], highlighting the cell type that was used for DNA methylation and chromatin accessibility analysis in (b-c).

(b) DNA methylation (yellow) and chromatin accessibility (green) profiles quantified over multiple genomic contexts in WT and *Tet*-TKO cells. Each panel corresponds to a different cell type: Surface ectoderm (top), ExE mesoderm (middle), and Pharyngeal mesoderm (bottom). Each column corresponds to a different genomic context: Promoters (N=18,329), Surface ectoderm enhancers (N=1,422), ExE mesoderm enhancers (N=3,178) and Pharyngeal mesoderm enhancers (N=982). Shown is the mean +/- 1 standard deviation in running averages of 50bp windows around the centre of the genomic annotation (2kb upstream and downstream).

(c) Box plots show the distributions of DNA methylation (top row, yellow) and chromatin accessibility (bottom row, green) in WT and *Tet*-TKO cells at different genomic annotations (columns). Each panel corresponds to a different cell type: Surface ectoderm (top), ExE mesoderm (middle), and Pharyngeal mesoderm (bottom).

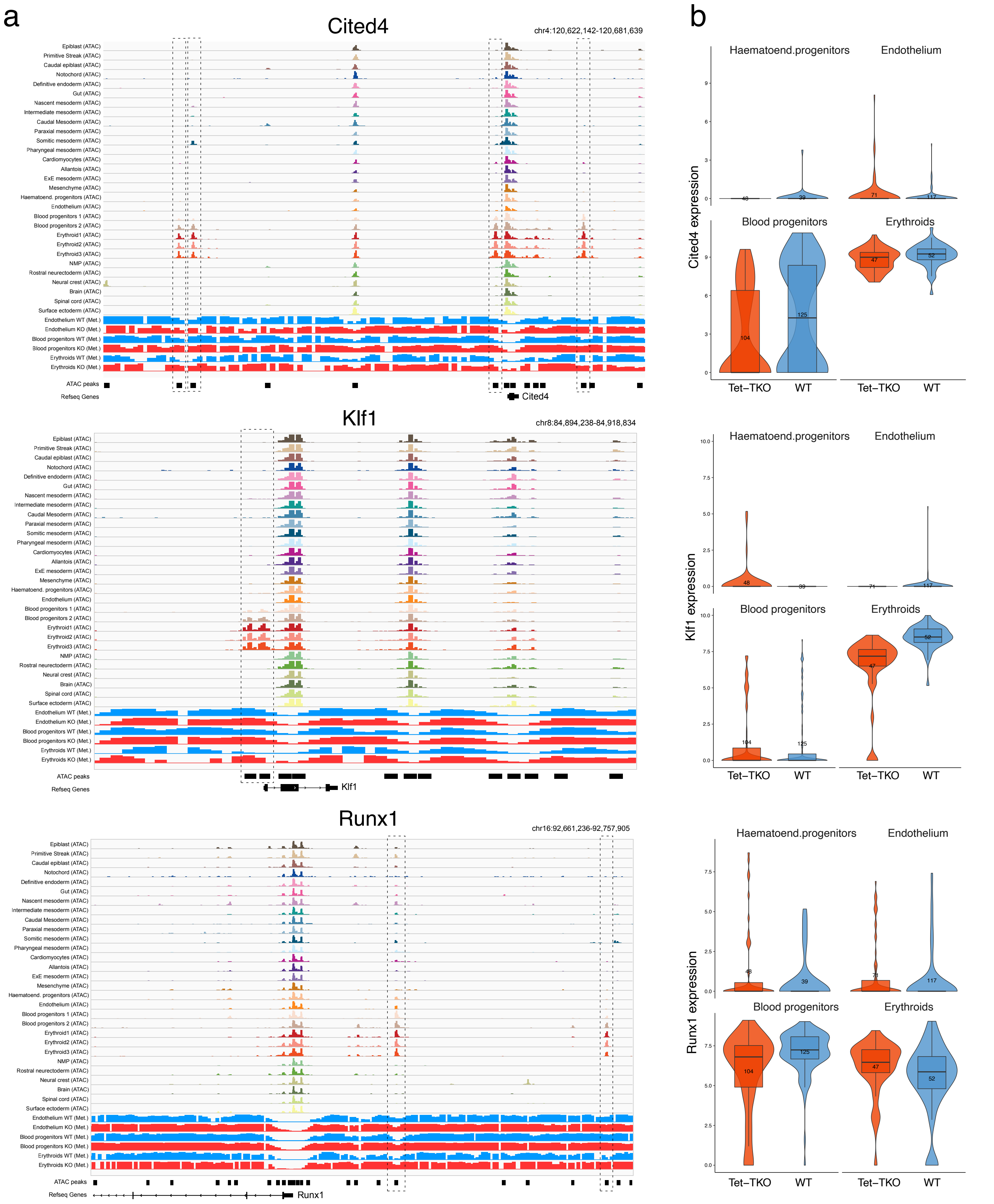

Figure S14: Examples of individual cis-regulatory regions that are dsyregulated in *Tet*-TKO cells.

(a) Genome browser plots of loci containing differentially expressed genes between WT and Tet-TKO cells at hematopoietic cell types. Shown is the ATAC-seq coverage across cell types from the reference data set and the DNA methylation coverage in the scNMT-seq data set, grouped by WT (blue) and KO (red). Highlighted with dashed lines are cis-regulatory regions identified from the ATAC-seq that are differentially methylated between WT and Tet-TKO cells and are linked to genes that are differentially expressed between WT and Tet-TKO cells at hematopoietic cell types.

(b) RNA expression values of the same genes as in (a), grouped by cell type.
